# Protein structure prediction via deep learning: an in-depth review

**DOI:** 10.3389/fphar.2025.1498662

**Published:** 2025-04-03

**Authors:** Yajie Meng, Zhuang Zhang, Chang Zhou, Xianfang Tang, Xinrong Hu, Geng Tian, Jialiang Yang, Yuhua Yao

**Affiliations:** ^1^ College of Computer Science and Artificial Intelligence, Wuhan Textile University, Wuhan, China; ^2^ Geneis Beijing Co, Beijing, China; ^3^ School of Mathematics and Statistics, Hainan Normal University, Haikou, China; ^4^ Key Laboratory of Data Science and Intelligence Education, Ministry of Education, Hainan Normal University, Haikou, China; ^5^ Key Laboratory of Computational Science and Application of Hainan Province, Hainan Normal University, Haikou, China

**Keywords:** protein structure prediction, deep learning, large language model, protein structure databases, evaluation index

## Abstract

The application of deep learning algorithms in protein structure prediction has greatly influenced drug discovery and development. Accurate protein structures are crucial for understanding biological processes and designing effective therapeutics. Traditionally, experimental methods like X-ray crystallography, nuclear magnetic resonance, and cryo-electron microscopy have been the gold standard for determining protein structures. However, these approaches are often costly, inefficient, and time-consuming. At the same time, the number of known protein sequences far exceeds the number of experimentally determined structures, creating a gap that necessitates the use of computational approaches. Deep learning has emerged as a promising solution to address this challenge over the past decade. This review provides a comprehensive guide to applying deep learning methodologies and tools in protein structure prediction. We initially outline the databases related to the protein structure prediction, then delve into the recently developed large language models as well as state-of-the-art deep learning-based methods. The review concludes with a perspective on the future of predicting protein structure, highlighting potential challenges and opportunities.

## 1 Introduction

**FIGURE 1:**
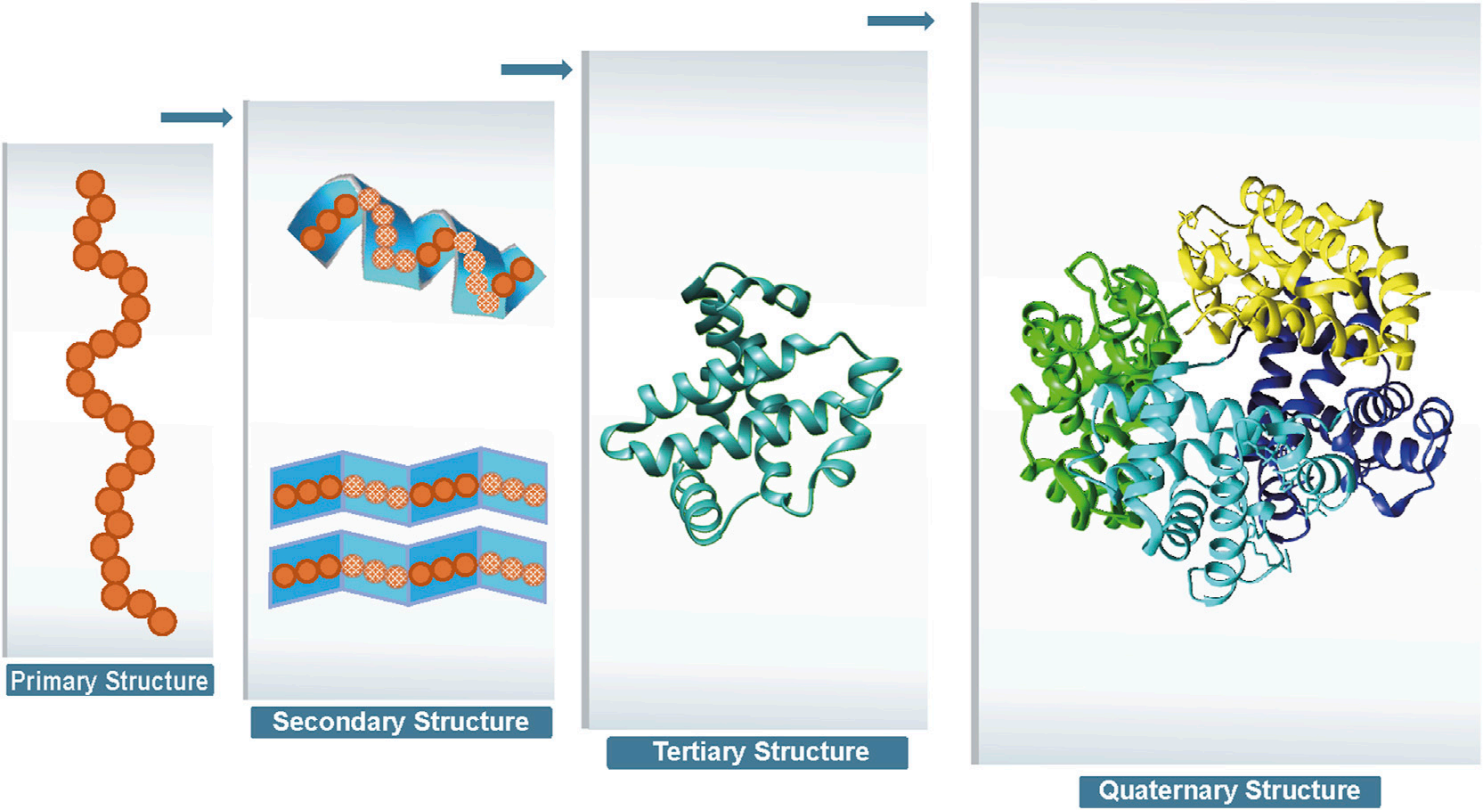
Four levels of protein structure.

**FIGURE 2:**
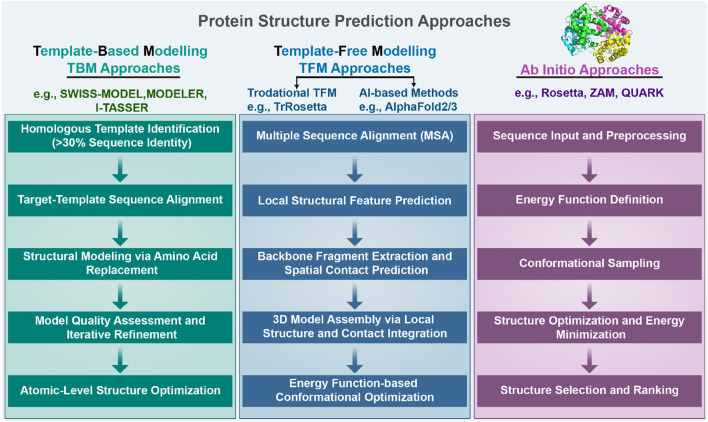
We categorize protein structure prediction approaches into three types: template-based modeling (TBM), template-free modeling (TFM), and *ab initio*. Detailed steps of these three approaches are provided in the Introduction section.

Proteins are one of the most important chemicals in animals and the material basis of living organisms. Proteins undertake various vital activities of living organisms, such as material transport, energy conversion, and catalytic reactions. A protein molecule is composed of several different amino acids, and there are 20 different types of amino acids that undergo dehydration condensation chemical reactions to form peptide bonds, which in turn form a sequence of amino acids linked from the beginning to the end. Then, transformations, such as helices, folding, and chemical reactions, result in the formation of proteins that are complex in both physical space and structure (Wang and Dunbrack Jr 2003). Protein structure can be divided into four levels, as shown in Figure 1. The primary structure of a protein is the linear sequence of amino acids, which is determined by the nucleotide sequence of the corresponding gene. The peptide chain results from dehydration condensation between amino acids to form peptide bonds. The number of polypeptide chains, the order of amino acid arrangement, and the number and positions of the bonds of peptide chains determine the primary structure of a protein. Hydrogen bonds are formed by the atoms between the residues of the peptide chain, which results in changes in the local structure of the peptide chain. Protein structure of proteins refers to the regular coiling or folding patterns formed by the polypeptide backbone in localized regions, which is stabilized by hydrogen bonds between backbone groups. Common secondary structural motifs include the alpha-helices and beta-sheet (Onofrio et al., 2014). The secondary structure is only related to the spatial position of the backbone atoms of the main chain and not to the position of the side chains (R groups). Protein tertiary structure is formed by the interaction of distant side chains in the protein secondary structure, and the three-dimensional (3D) spatial arrangement of the main chain and side chains after folding and coiling constitutes the protein’s tertiary structure. It gives rise to two major molecular shapes called fibrous and globular. Globular protein structures can be divided into four structural classes (i.e., main alpha-structure, main beta-structures, alpha/beta-structures, alpha + beta-structures). The function of a protein is largely determined by its tertiary structure, which fully describes its 3D shape. The function of a protein is largely determined by its tertiary structure, which fully describes its 3D shape. While the native state of globular proteins corresponds to a thermodynamically stable energy minimum under physiological conditions, pathological aggregates such as amyloids can occupy deeper energy minima stabilized by cross- β sheet interactions. This complexity in the energy landscape makes protein structure prediction particularly challenging (Majid and Khan, 2023). The quaternary structure of proteins refers to the architecture of a complex formed by two or more protein molecules, known as protein subunits, interacting through non-covalent bonds. The problem of protein structure prediction focuses on the transformation from amino acid sequence to protein 3D structure (Anfinsen, 1973). The problem of protein structure prediction focuses on the transformation from amino acid sequence to protein 3D structure. While Anfinsen’s dogma established that the native structure of a protein is determined by its amino acid sequence, the Levinthal paradox highlights a fundamental challenge in this process (Levinthal, 1968). Cyrus Levinthal pointed out that if a protein were to sample all possible conformations randomly to find its native structure, it would take an astronomically long time given the enormous number of possible conformations. However, proteins in nature fold reliably in microseconds to seconds. This paradox demonstrates the inherent complexity of the protein folding process, while simultaneously suggesting that protein folding must proceed along specific pathways rather than through random conformational searches. This theoretical framework has motivated scientists to develop a wide range of approaches for protein structure prediction. A comprehensive and in-depth analysis of the multitude of protein sequences, along with the mining of concealed information, holds profound significance in the fields of modern biology, medicine, and pharmaceuticals (Kuhlman and Bradley, 2019; Li et al., 2021). Due to the extremely rapid growth of protein data and the large scale of data, traditional experimental methods such as NMR and X-ray diffraction to obtain protein structures have the limitations of long cycle time, high cost, and high intermediate product requirements, and the rate of access to resolved protein structures using experimental methods is much slower than the explosive growth of protein sequences (Ladd et al., 1977; Sorgen, 2005). As of 2022, according to the TrEMBL database (Consortium, 2020), there are over 200 million sequence entries, with only 200,000 known protein structures according to the Protein Data Bank (PDB) database (Compton, 2003). It is not feasible to extract protein structure information from experimental methods alone, and a method that enables rapid and accurate prediction of protein structure based on amino acid sequence information needs to be explored. Protein structure prediction approaches can be classified into three categories: template-based modeling (TBM), template-free modeling (TFM), and *ab initio*. First, TBM approaches rely on identifying and using known protein structures as templates, typically through sequence or structural homology. Second, TFM approaches encompass both traditional (e.g., TrRosetta) and modern AI-based approaches (e.g., AlphaFold3). While commonly referred to as “template-free”, the modern AI-based approaches still rely heavily on comparative analysis and training data from the Protein Data Bank (PDB). It is important to emphasize that current AI-based approaches do not explicitly use templates, but their models are indirectly dependent on known structural information, as they are trained on large-scale PDB data. Despite their remarkable success, these AI-based tools show significant limitations when predicting structures of proteins that lack homologous counterparts in the PDB. Finally, the third category, *ab initio*, represents the true “free modeling” approach. Unlike TBM and TFM, *ab initio* approaches are based purely on physicochemical principles and do not rely on existing structural information. The specific steps involved in the three protein structure prediction approaches are illustrated in Figure 2. TBM tools is well represented by MODELLER Webb and Sali (2016) and SwissPDBViewer Guex and Peitsch (1997), where MODELLER implements multi-template modeling to integrate local structural features from multiple homologous templates, while SwissPDBViewer provides comprehensive tools for protein structure visualization and analysis. TBM involves comparing the target sequence with a suitable template structure and then selecting the model with the best match while considering mutations, deletions, and insertions that may be present in the target template structure (Kong et al., 2021; Wu and Xu, 2021; Kong et al., 2022; Weißenow et al., 2022; Sun et al., 2013). The specific steps are as follows. Step 1 involves identifying a homologous protein structure that serves as a template for the target protein. It is crucial that the target sequence and the template sequence share a sequence identity of at least 30%. Step 2 entails creating a sequence alignment between the target sequence and the template sequence. This alignment lays the foundation for accurately mapping the amino acids from the target sequence to their corresponding positions in the template structure. In step 3, through the sequence alignment, amino acids from the target sequence are replaced into the spatial positions of corresponding amino acids in the template structure. This replacement and modeling process is facilitated by homology modeling software, which utilizes the alignment to predict the three-dimensional structure of the target protein. Step 4, the generated structural model undergoes a quality assessment to evaluate its accuracy and reliability. Based on the assessment results, the sequence alignment may be adjusted or corrected, followed by a reiteration of the model building process. This cycle of model building and quality evaluation continues until the model meets the required quality standards. Finally, in step 5, the 3D structure is then refined at the atomic level to obtain the final predicted model. TBM is based on the distance between the target protein structure and the template protein structure, which can be subdivided into comparative modeling and threading. Comparative modelling, also known as Homology modeling, is designed for target proteins with near-homologous templates, and templates can be usually identified by sequence-based comparisons. Threading, also known as fold recognition, operates under the premise that dissimilar amino acid sequences can map onto similar protein structures. Protein threading involves comparing a target sequence andor a hidden Markov model Karplus (2009) against one or more protein structures to identify the best matching sequence-structure template pair. Consequently, threading can effectively identify similar folds or structural motifs in a target sequence, even when sequence similarity is minimal. As different threading programs are trained with different scoring functions and matching algorithms, the template recognition and matching results are often different for the same query sequence. However, establishing the best sequence–template pairing is very challenging, especially when only remotely related templates to the target protein are available.

TFM predicts the structure of a protein directly from the sequence without using global template information by using only amino acid sequence information and without reference to any protein template (Xu and Wang, 2019; Senior et al., 2019; de Oliveira et al., 2021; Hou et al., 2019; Chen et al., 2020). The steps for this process are delineated as follows. Step 1 involves performing multiple sequence alignments (MSAs) between target proteins and their homologous sequences. This process gathers information about amino acid alterations between the homologous sequences and discerns correlation patterns of sequence changes occurring at varied positions. Step 2: Target protein sequences and multiple sequence comparisons are used to construct the basis for predicting local structural frameworks, including torsion angles and secondary structures. Step 3: Backbone fragments are extracted from proteins predicted to have similar local structures and are used for model building, and based on the mutations in multiple sequence comparisons, residue pairs that may be in spatial contact can also be predicted. Step 4: 3D models of protein structures are built by prediction of local structure and disability contacts, which includes gradient-based optimization, distance geometry, and fragment assembly. Step 5: Based on the large search space, the model is improved using the energy function to identify low-energy conformational groups by comparing them with each other. Given the water-soluble nature of amino acids in proteins, physically standard molecular dynamics potential energy functions are used to model protein folding; the protein structure is most stable when the energy is at its lowest. Fragment assembly, a highly effective approach in free modeling, starts by identifying short structural fragments from unrelated proteins. Fragment lengths can be discrete or continuous, and the fragments are mainly based on the comparison of local structural features extracted from the template, such as secondary structure, solvent accessibility, twist angle, and other similarities. Fragment assembly simulation is performed by replacing the main chain structure of a specific region of the simulated structure with the structure of the selected fragment, which can be of the desired bond length, angle, or other component. The replaced fragment can be extracted directly from the fragment itself. Constructing models through fragment assembly reduces the entropy of the conformational search space while ensuring that the local structure of the model is well-formed. TFM is more time-consuming than TBM, as it requires the creation of a model from a random conformation. Although some optimization algorithms such as gradient descent have made progress, there is still a disparity between TFM and TBM in terms of accuracy.

Ab initio approaches rely entirely on physicochemical principles (such as molecular mechanics force fields and energy minimization) and conformational search algorithms, without depending on any known structural data (including training data) for prediction (Pierri et al., 2008). This approach is based on Anfinsen’s thermodynamic hypothesis, which states that a native structure corresponds to the global free energy minimum under a given set of conditions. Among the notable *ab initio* tools, Rosetta (Rohl et al., 2004) employs a technique called Monte Carlo with Minimization to explore the conformational space of a protein and predict its three-dimensional structure from the amino acid sequence. This method iteratively optimizes to find the lowest energy conformation. QUARK (Xu and Zhang, 2012) stands as another representative tool, specifically designed for *ab initio* protein structure prediction and peptide folding simulation. QUARK models are built from small fragments (1–20 residues long) by replica-exchange Monte Carlo simulation under the guidance of an atomic-level knowledge-based force field. Despite its capabilities, the tool is constrained by computational limitations, being applicable only to proteins shorter than 200 amino acids and requiring over 48 h for structure prediction. While these approaches are computationally expensive and have certain limitations, they offer unique value in understanding the fundamental physical principles of protein folding, particularly for novel proteins lacking homologous templates in structural databases (Dill et al., 2008).

The structure of this review is as follows. Section 1 describes the generation of protein structures and the two types of modeling approaches currently available for protein structure prediction. Section 2 articulates the necessity for protein structure prediction and explores the potential influence of deep learning in this domain. Section 3 lists publicly available databases in the field. Section 4 discusses in detail the contribution of deep learning in this area of study. Section 5 summarizes our work, offering a perspective on the potential future directions of the field.

## 2 Current status of protein structure prediction research

The determination of protein structures has led to a greater understanding of the foundations of biology. Physical experiments such as X-ray crystallography, nuclear magnetic resonance spectroscopy, and cryo-electron microscopy have helped obtain protein structures (Ladd et al., 1977; Sorgen, 2005; Cheng et al., 2021), but there is still a large and growing gap between the number of proteins and the number of known protein structures. The study of protein structure is necessary, and protein structure prediction is an important area of research in biology. For example,: protein structure prediction is a crucial part of protein design. It has been widely used for the evaluation of designed candidate proteins with topology or symmetry constraints (Huang and Li, 2023). The pathological features of some diseases are also related to proteins, the two primary pathological features of Alzheimer’s disease are the accumulation of Amyloid-beta (Aβ) plaques and hyperphosphorylated tau (p-tau) protein, which form neurofibrillary tangles (Lin R.-R. et al., 2023). When an organism is infected by microorganisms such as parasites, bacteria, or viruses, certain proteins play a key role in the immune response by acting as antibodies. These proteins are involved in detecting and neutralizing the pathogens, helping the organism defend against the disease. When an organism is treated with a drug, specific proteins can serve as target receptors. The drug molecule interacts with these receptors, allowing the drug to bind to the correct protein in the body, thereby triggering the appropriate chemical reaction and producing the desired therapeutic effect to treat the disease. In this regard, the prediction of protein structure is a prerequisite for research on drug reuse, disease treatment, and protein function (Pan et al., 2022; Gligorijević et al., 2021; Xu et al., 2022; Tang et al., 2021). As a basis for the prediction of drug–target, drug–disease (Yang et al., 2020b; Liu et al., 2020; Meng et al., 2022), and target–disease associations in the study of protein function and drug repositioning and based on the widening gap between the number of known proteins and the number of actual proteins, protein structure prediction requires more powerful deep learning for research. Artificial intelligence (AI) is broadly affecting many aspects of various fields and addressing diverse tasks and problems in place of humans (Huang et al., 2023; Fu et al., 2024). Deep learning is gradually becoming a key technique in research areas such as computer vision, speech recognition, and natural language processing (Esteva et al., 2021, Chai et al., 2021; Cochero et al., 2022; Santhanavijayan et al., 2021; Shahamiri, 2021; Alsayadi et al., 2021; Pandey et al., 2022; Lauriola et al., 2022; Wahab et al., 2021; Yang et al., 2022; Ye et al., 2022; Li et al., 2022). With advances in deep learning algorithms and increased computing power, great progress has been made in biomedical fields such as predicting protein structures, single-cell technologies (Wen et al., 2022), and cancer research (Gu et al., 2020; Ji et al., 2023; Shi et al., 2022). CASP is an international competition to assess the state of the art in modeling protein structures from amino acid sequences, with the aim of advancing the problem of computing 3D structures of proteins from amino acid sequence information (Qian et al., 2018). With the development of deep learning techniques, more than half of the teams involved in CASP 14 used deep learning algorithms in the protein structure prediction task, we employ data analysis of submissions to the CASP14 and CASP15 competitions as evidence. Our findings indicate that within CASP14, a total of 88 papers explicitly reported the use of deep learning methodologies, compared to 40 papers that did not incorporate such technologies. Similarly, in CASP15, 68 submissions were identified as utilizing deep learning approaches, whereas 16 submissions were found to abstain from applying deep learning techniques. The DL-based AlphaFold2 in CASP14 can accurately predict the 3D structures of 98.5% of human proteins. It is even considered to be the second-largest breakthrough in life sciences after the human genome project (Xu Y. et al., 2021). This demonstrates the excellent learning capability of deep learning and accelerates the development of the field of bioinformatics.

AlphaFold3, further extends these capabilities by modeling interactions between proteins and diverse biomolecules (e.g., DNA, RNA, ligands) with atomic precision (Abramson et al., 2024), demonstrating remarkable success in predicting protein complexes and multi-domain assemblies. However, the performance of AI-based tools like AlphaFold is inherently constrained by the limitations of the PDB. Recent studies highlight that the PDB’s restricted size and structural bias may lead to overfitting and memorization effects in deep learning models. Chakravarty et al. (2024) While AlphaFold3 improves upon its predecessor in capturing biomolecular interactions, it still struggles with dynamic systems such as fold-switching proteins. For example, AlphaFold3 failed to predict the experimentally observed dimeric conformation of human XCL1, instead generating a domain-swapped structure inconsistent with evolutionary restraints. This underscores a critical issue: the PDB predominantly contains static, thermodynamically stable conformations, with limited representation of dynamic or multi-state proteins. Consequently, even advanced models exhibit modest success rates for known fold-switching cases within their training sets and perform poorly on novel conformations These limitations emphasize that current AI tools remain dependent on the structural diversity present in training data, calling for expanded databases with transient states and hybrid approaches integrating physical modeling.

## 3 Summary of databases

The exponential growth of protein-related data, driven by advancements in genome sequencing and proteomic techniques, presents significant opportunities for computational protein structure prediction methods to reveal novel protein structures. The Protein Data Bank wwPDB consortium (2018) (PDB) serves as a repository for experimentally determined 3D structures, primarily focusing on proteins, nucleic acids, and biological macromolecules. As of 2023, the PDB contains an impressive collection of 214,108 structures. The Universal Protein Resource (UniProt) Consortium U. (2019) is a comprehensive database that offers detailed information on protein sequences and functional annotations. It consists of three databases: UniProtKB Boutet et al. (2007), UniParc, and UniRef. Among these, UniProtKB is the largest component, providing known protein sequences and related annotation information. UniParc serves as an archive library, housing copies of all known protein sequences, while UniRef is a protein clustering database that groups similar proteins, offering representative sequences and related annotations. Table 1 summarizes the databases involved in protein structure prediction.

**TABLE 1:** The databases involved in protein structure prediction.

Type	Name	Description	URL	API
*In vitro* determined structure	PDB Berman et al. (2000)	PDB is an archive of experimentally-determined structures of proteins, nucleic acids, and complex assemblies	www.rcsb.org	Yes
	MMDB Madej et al. (2014)	MMDB contains experimentally resolved structures of proteins, RNA, and DNA, derived from the PDB, with value-added features	www.ncbi.nlm.nih.gov/Structure/MMDB/mmdb.shtml	Yes
	SCOP Murzin et al. (1995)	A database of protein structural domains based on their evolutionary and structural relationships	https://scop.mrc-lmb.cam.ac.uk/	No
	BMRB Ulrich et al. (2007)	A repository for experimental and derived data of biomolecular NMR studies	https://bmrb.io/	Yes
In silico predicted structure	AlphaFold Protein Structure Database Jumper et al. (2021)	This platform provides open access to over 200 million protein structure predictions, aiming to accelerate scientific research	https://alphafold.ebi.ac.uk/	No
Sequence	UniProt Consortium (2019b)	A comprehensive protein sequence and functional annotation database, consisting of UniProtKB, UniParc, and UniRef.	www.uniprot.org/	Yes
	ModBase Pieper et al. (2014)	A database of comparative protein structure models based on the sequences of proteins with known structures	https://modbase.compbio.ucsf.edu/	No
	ProteinNet AlQuraishi (2019)	A standardized data set for machine learning of protein structure	https://github.com/aqlaboratory/proteinnet	No
	ENA Amid et al. (2020)	ENA provides a comprehensive record of the world’s nucleotide sequencing information, covering raw sequencing data, sequence assembly information and functional annotation	www.ebi.ac.uk/ena	Yes
	GenBank Benson et al. (2012)	A database of nucleotide sequences that is maintained by the National Center for Biotechnology Information (NCBI)	www.ncbi.nlm.nih.gov/genbank/	Yes
	HSSP Dodge et al. (1998)	A database of alignments of protein sequences and their secondary structures, which can be used for the prediction of protein structures	https://swift.cmbi.umcn.nL/gv/hssp/	No
Family	Pfam Mistry et al. (2021)	It is a comprehensive collection of protein domains and families, represented as multiple sequence alignments and as profile hidden Markov models	https://pfam.xfam.org/	Yes
	HOMSTRAD Mizuguchi et al. (1998)	A curated database of protein structure alignments for homologous families	www-cryst.bioc.cam.ac.uk/homstrad/	No
	SUPERFAMILY Gough and Chothia (2002)	A database of protein domains and their relationships, based on hidden Markov models (HMMs) and structural alignments	https://supfam.org/	No
	PROSITE Sigrist et al. (2010)	A database of protein families, domains, and functional sites, which are annotated with information about their structure, function, and evolutionary history	https://prosite.expasy.org/	NO
Family	InterPro Mitchell et al. (2015)	A database of protein families, domains, and functional sites, which integrates information from several different databases and predictive algorithms	www.ebi.ac.uk/interpro/	Yes
	SMART Letunic et al. (2015)	A database of protein domains and families, providing information about the sequence, structure, and function of these domains and families	https://smart.embl-heidelberg.de/	Yes
Interaction	DIP Salwinski et al. (2004)	Catalogs experimentally determined interactions between proteins	https://dip.doe-mbi.ucla.edu/	No
	STRING Szklarczyk et al. (2019)	A database of protein-protein interactions and functional associations, which integrates experimental and computational data from multiple sources	https://string-db.org/	Yes
	BioGRID Oughtred et al. (2019)	A database of protein-protein and genetic interactions, integrating experimental data from high-throughput screens and literature curation	https://thebiogrid.org/	Yes
Function	CATH Sillitoe et al. (2021)	CATH is a hierarchical classification of protein domains based on their structures and functions	www.cathdb.info/	No
	PIR Wu et al. (2002)	An integrated public resource of functional annotation of protein data to aid in the exploration of the protein universe	https://pir.georgetown.edu/	Yes
Hybrid	NKAB Berman et al. (2022)	This tool offers searching, reporting, statistical analysis, mapping, and visualization for all experimentally determined 3D structures involving nucleic acids, maintained by both NDB and PDB.	https://nakb.org/	No
	SWISS-MODEL Waterhouse et al. (2018)	A web-based protein structure homology-modeling server that uses a template-based approach to generate three-dimensional models of proteins	swissmodel.expasy.org/	Yes
	PSPC Moult et al. (2018)	It provides resources for the community-wide Critical Assessment of Techniques for Protein Structure Prediction (CASP) experiments	https://predictioncenter.org/	No
	EVcouplings Hopf et al. (2019)	A platform for predicting protein structure, function, and mutations using evolutionary sequence covariation	https://evcouplings.org/	No
	PMP Haas et al. (2013)	PMP provides a single, consistent interface to various sources of computational models of protein structure	https://proteinmodelportal.org/	Yes
	PRISM server Baspinar et al. (2014)	A server for predicting protein-protein interactions and modeling their 3D complexes	https://prism.ccbb.ku.edu.tr/	No
	SGC Lee et al. (2009)	A not-for-profit organization that aims to determine the three-dimensional structures of proteins of medical relevance	www.thesgc.org/	No
	Protein Atlas Thul and Lindskog (2018)	It aims to map all the human proteins in cells, tissues and organs using integration of various omics technologies	www.proteinatlas.org/	Yes
	COFACTOR Zhang et al. (2017)	A protein function prediction tool that uses multiple sources of data to generate predictions, including protein-protein interaction data, gene ontology terms, and sequence information	https://zhanglab.ccmb.med.umich.edu/COFACTOR/	No
	HMMER Finn et al. (2011)	It is a tool for searching protein sequences against a database of HMMs of protein families and domains	https://hmmer.org/	Yes
Hybrid	Robetta Kim et al. (2004)	A protein structure prediction server that uses comparative modeling, *de novo* modeling, and structure-based protein function prediction	https://robetta.bakerlab.org/	No
	MODELLER Webb and Sali (2016)	A software package for protein structure prediction, which uses comparative modeling to generate 3D models of protein structures based on homology to known structures	https://salilab.org/modeller/	Yes
	Phyre2 Kelley et al. (2015)	A web-based service for protein modeling, prediction, and analysis	www.sbg.bio.ic.ac.uk/phyre2	No
	PconsFold Yang et al. (2020a)	A tool for protein structure prediction, which uses a probabilistic approach to generate models that are more accurate and reliable than those generated by traditional methods	https://toolkit.tuebingen.mpg.de/tools/pcons-fold	Yes
	ModFOLD McGuffin (2008)	A tool for protein structure prediction, which uses an ensemble approach to generate models that are more accurate and reliable than those generated by individual methods	www.reading.ac.uk/bioinf/ModFOLD/	Yes
	PIP Ofran and Rost (2003)	A tool for protein-protein interaction prediction, which uses a machine learning approach to predict the interaction partners of a query protein based on its sequence and structural features	www.pip-tools.org/	Yes
	MolIDE Canutescu and Dunbrack Jr (2005)	A tool for interactive molecular visualization and analysis, providing a user-friendly interface for exploring protein structures and their interactions	https://dunbrack.fccc.edu/molide/molide.php	No
	PRODIGY Xue et al. (2016)	A tool for predicting protein-ligand binding affinity, using a physics-based approach to model the thermodynamics and kinetics of the binding process	https://milou.science.uu.nL/services/PRODIGY/	Yes
	Reactome Fabregat et al. (2018)	A bioinformatics tool for visualizing, interpreting, and analyzing pathway knowledge	https://reactome.org/	Yes
	HHblits Remmert et al. (2012)	A tool for protein sequence alignment, using a profile hidden Markov model (HMM) to align query sequences against a database of HMMs for protein families and domains	https://toolkit.tuebingen.mpg.de/tools/hhblits	Yes
	PISA server Krissinel and Henrick (2007)	A server for analyzing protein-protein and protein-ligand interactions, providing information about the geometry, energetics, and surface area of these interactions	www.ebi.ac.uk/pdbe/pisa/	Yes
	CRISPR Grissa et al. (2007)	A database of CRISPR/Cas systems, providing information about the classification, function, and diversity of these systems in bacteria and archaea	https://crispr.i2bc.paris-saclay.fr/	No

Accessing publicly available datasets is essential for leveraging data in deep learning models. Therefore, ensuring easy downloads or APIs for dataset availability is crucial. Researchers have the flexibility to select inputs from diverse data sources and conduct cross-database comparative analyses. Protein structure prediction, a complex and challenging task, requires a range of databases. These databases can be broadly categorized into six types: protein sequence, structure, family, interaction, function, and hybrid methods databases. The PDB exemplifies a protein structure database, offering experimentally determined 3D structures of proteins (Burley et al., 2017). The quality and quantity of structural databases directly determine the level of development and optimization of structural prediction methods. More structural data allows for more accurate algorithm training and testing, thereby improving the accuracy of predictive models. However, it’s also important to consider complementary datasets providing sequence information, protein associations, family details, and functional annotations. Sequence databases like UniProt Consortium U. (2019) and RefSeq O’Leary et al. (2016) database at NCBI contain amino acid sequences of numerous proteins, serving as the foundation for protein structure prediction. The breadth and depth of sequence databases directly affect the accuracy and feasibility of protein structure prediction. More abundant sequence data means a higher chance of finding sequences highly similar to the target protein, thereby increasing the success rate of structure prediction. Family databases classify proteins based on sequence and structural similarities. By providing structural information of similar family members, the family database supports accurate model prediction on unknown proteins. In addition, the functional information of the family database provides strong support for annotation and prediction of protein functions. The widely used Pfam database focuses on protein families, while InterPro(Paysan-Lafosse et al., 2023) integrates multiple databases, including Pfam, ProSite Sigrist et al. (2012), and PRINTS Attwood et al. (2000). Additionally, protein family databases like SMART (Letunic et al., 2012), CDD (Lu et al., 2020), and PROSITE Sigrist et al. (2012) greatly contribute to understanding protein structure-function relationships. Interaction databases such as STRING Szklarczyk et al. (2021), I2D Brown and Jurisica (2005) and BioGrID Oughtred et al. (2021) provide valuable information on protein-protein interactions, including functional and regulatory associations. Interaction information enriches the context of structure prediction, allowing researchers to not only predict the structure of individual proteins but also to predict the interactions and arrangements of proteins within complexes. Function databases like CATH Sillitoe et al. (2021), PIR Wu et al. (2002) and Gene Ontology Consortium G. O. (2019), aid in comprehending the connection between protein structure and function. Functional information provides important clues for predicting structures, especially in predicting protein functional domains and active sites, helping to improve the relevance and accuracy of structure predictions. Hybrid methods databases, including ModBase (Pieper et al., 2014), Robetta Kim et al. (2004), and SWISS-MODEL Waterhouse et al. (2018), offer integrated tools and resources that combine multiple approaches. The comprehensive information from hybrid databases makes structure prediction more holistic and accurate, especially for complex prediction tasks that require an integrated consideration of sequence features, structural patterns, and functional information.

## 4 Advances in deep learning for protein structure prediction

Machine learning techniques have contributed substantially to the generation of innovative concepts in the field of protein structure prediction, resulting in notable advancements. Most machine learning methods for protein structure prediction have focused on methods based on co-evolution (Bonetta and Valentino, 2020; S Bernardes, 2013; Zhang and Zhang, 2019; Yang et al., 2014). The accuracy of these methods depends on the number of homologous protein sequences available in the database. Protein structure prediction is challenging when there are no target proteins with homologous protein sequences in the database. Machine learning models with simpler structures are unable to predict them accurately, whereas deep learning can learn deeper and more complex structural features; thus, deep learning models are considered for protein structure prediction. Deep learning methods can be utilized to integrate and extract features from these various data sources, allowing for accurate and efficient prediction of protein structures. Table 2 summarizes the deep learning models used in the protein structure prediction. Table 3 lists the available online web servers for protein structure prediction. Integrating data from multiple sources can lead to more accurate predictions of protein structure and function. One example is the AlphaFold algorithm, which combines PDB data, protein sequence data from UniProt, and multiple sequence alignment data from publicly available databases. The utilization of these data sources in combination with deep learning approaches has led to significant advancements in the field of protein structure prediction, offering new avenues for drug discovery and protein engineering. We next analyze and summarize deep learning models such as deep neural networks, convolutional neural networks, recurrent neural networks, graph neural networks, and deep residual neural networks for protein structure prediction.

**TABLE 2:** Models used for protein structure prediction.

Model	Input	Architecture	Year	URL
SPIN2 O’Connell et al. (2018)	Sequence	DNN	2018	https://sparks-lab.org
MULTICOM Hou et al. (2020)	Sequence	DNN	2020	https://github.com/multicom-toolbox/multicom/
APPTEST Timmons and Hewage (2021)	Sequence	CNN	2021	https://research.timmons.eu/apptest
ProALIGN Kong et al. (2022)	Sequence, Secondary Structure	CNN	2022	NA
2C-BRNN Guo et al. (2018)	Sequence	RNN	2018	https://github.com/guoyanb/JBCB 2018/
CRNN Zhong and Gu (2020)	Sequence	RNN	2020	NA
CSI-LSTM Miao et al. (2021)	Sequence, Secondary Structure	LSTM	2021	https://github.com/eagleccnu/CSI_LSTM/tree/master
PG-GNN Xia and Ku (2021)	Sequence	GNN	2021	NA
Nahid et al. Nahid et al. (2021)	Sequence	GNN	2021	NA
DeepMetaPSICOV Kandathil et al. (2019)	Sequence	ResNet	2019	https://github.com/psipred/DeepMetaPSICOV/
Yang et al. Yang et al. (2020a)	MSA	ResNet	2020	https://github.com/gjoni/trRosetta
ThreaderAI Zhang and Shen (2020)	Sequence	ResNet	2020	https://github.com/ShenLab/ThreaderAI
ProSPr Stern et al. (2021)	Sequence, MSA	ResNet	2021	https://github.com/dellacortelab/prospr
Xu et al. Xu et al. (2021a)	Sequence	ResNet	2021	https://github.com/j3xugit/RaptorX-3DModeling/
NDThreader Wu and Xu (2021)	Sequence	ResNet	2021	https://github.com/wufandi/DL4SequenceAlignment
Alphafold2 Jumper et al. (2021)	Sequence, MSA	Transformer-based	2021	https://github.com/deepmind/alphafold
RoseTTAFold Baek et al. (2021)	Sequence, MSA	Transformer-based	2021	https://github.com/RosettaCommons/RoseTTAFold
trRosetta Du et al. (2021)	Sequence	Transformer-based	2021	https://yanglab.nankai.edu.cn/trRosetta/
RGN2 Chowdhury et al. (2022)	Sequence	Transformer-based	2022	https://github.com/aqlaboratory/rgn2/
ESMfold Lin et al. (2023b)	Sequence	Transformer-based	2023	https://github.com/facebookresearch/esm
ProteiNN Szelogowski (2023)	Sequence	Transformer-based	2023	https://github.com/danielathome19/ProteiNN-Structure-Predictor/
AlphaLink Stahl et al. (2023)	Sequence, MSA	Modified AlphaFold2	2023	https://github.com/lhatsk/AlphaLink/
Alphafold3 Abramson et al. (2024)	Sequence, Ligands, covalent bonds	Transformer-based	2024	https://github.com/google-deepmind/alphafold3
EigenFold Jing et al. (2023)	Sequence	Diffusion-based	2023	https://github.com/bjing2016/EigenFold/
RFdiffusion Watson et al. (2023)	Sequence	Diffusion-based	2023	https://github.com/RosettaCommons/RFdiffusion/
OmegaFold Wu et al. (2022)	Sequence	Transformer-based, LLM	2022	https://github.com/HeliXonProtein/OmegaFold

**TABLE 3:** Web servers available for protein structure prediction.

Web server	URL
AlphaFold3	https://alphafoldserver.com/
D-I-TASSER	https://zhanggroup.org/D-I-TASSER/
Robetta	https://robetta.bakerlab.org
I-TASSER	https://zhanggroup.org/I-TASSER/
Phyre2	http://www.sbg.bio.ic.ac.uk/phyre2/html/page.cgi?id=index
Modeller	https://salilab.org/modeller/
SWISS-MODEL	https://swissmodel.expasy.org
ModeBase	https://modbase.compbio.ucsf.edu/modweb/
DMFold	https://zhanggroup.org/DMFold/

### 4.1 Deep neural networks

**FIGURE 3:**
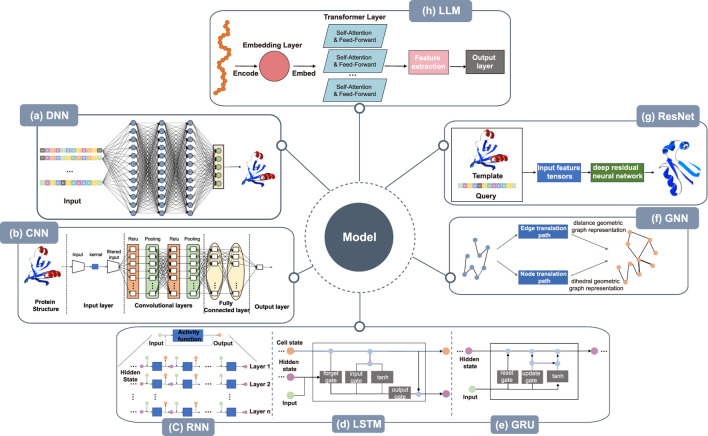
Architecture of deep learning models. **(a)** DNN takes the protein sequence as input and outputs the protein structure after processing through several hidden layers. **(b)** The CNN takes the protein structure as input for pre-processing, then, within the convolution layer, features are extracted by convolutional operations to reduce noise and pool data features remain unchanged while the data is compressed to reduce overfitting. After several rounds of convolution and pooling operations, the data is compressed. At the same time, the data is abstracted into features with higher information content, and finally, through the fully connected layer, the results are obtained. **(c)** RNN takes protein sequence data as input and increases the number of layers of the network for vertical expansion, using chaining and recursion to finally obtain prediction results. **(d)** LSTM can solve the long-term dependency problem found in general RNN, as well as issues such as long-term memory and gradients in back propagation. **(e)** GRU, a variation of LSTM, runs more efficiently than LSTM networks. GRU can achieve comparable results and can improve training efficiency to a great extent. **(f)** The amino acid sequence of the protein is used as input in GNN to abstract the protein structure as a graph structure. The features of nodes and edges are extracted by edge embedding and node embedding to obtain edge translation path and node translation path. In node translation path, each amino acid is considered as a node within a graph, with the node’s feature vector typically encompassing the physicochemical properties of the amino acid. The translation of edges focuses on the interactions between amino acids in the protein sequence. In GNNs, edges represent the relationships between nodes (amino acids), and by updating the weights of these edges, it’s possible to capture these interactions, thereby reflecting the three-dimensional structural characteristics of proteins in the graph. The geometry of the 3D protein backbone structure is then predicted after the distance geometric graph representation and the dihedral geometric graph representation, respectively. **(g)** Deep residual neural network takes protein templates and query sequences as input and predicts the protein 3D structure by the input feature tensor. **(h)** Large language models train a processed data, often using techniques like transfer learning from pre-trained models.

Deep neural networks (DNNs) are also called multilayer perceptrons. The layers within a DNN can be divided into three categories: the input layer, the hidden layer, and the output layer, with nodes fully connected between the layers. The framework of the model is shown in Figure 3a. The amino acid sequence is input to the DNN after one-hot encoding or embedding representation, and after the processing through the hidden layers, a number of protein structures are finally output, with the structure having the highest score as the final prediction.DNNs have been used by many researchers to model protein structure prediction. Aaron Hein et al. used artificial neural networks (ANNs) to optimize the encoding of protein primary sequence structure, which helps in the prediction of protein secondary structure and protein tertiary structure, thus improving the quality of protein structure prediction Hein et al. (2021). John Jumper et al. designed important DNN-based protein 3D structure model, called MULTICOM. MULTICOM is an automated protein structure prediction system that involves three major components: contact distance prediction based on deep convolutional neural networks, distance-driven template-free modeling, and protein model ranking that’s empowered by deep learning and contact prediction (Hou et al., 2019; Hou et al., 2020; Wang et al., 2010). In general, deep neural networks can assist in predicting protein primary, secondary, and tertiary structures. These networks have shown promise in optimizing predictions for primary and secondary structures (Senior et al., 2019; Du et al., 2021; Timmons and Hewage, 2021; Senior et al., 2020; Mulnaes et al., 2020; Ju et al., 2021). While deep neural networks (DNNs) have demonstrated remarkable success in predicting secondary and tertiary structures of globular proteins, These methods excel when evolutionary or structural homologs exist in training datasets (e.g., the PDB), leveraging coevolutionary patterns to infer folds. However, their efficacy diminishes for non-globular proteins, such as intrinsically disordered proteins (IDPs) or fold-switching systems, where training data are sparse or conformational diversity is critical. For example, AlphaFold often mispredicts alternative folds or dynamic conformations due to overreliance on static training-set structures (Chakravarty et al., 2025). Similarly, DNNs struggle with membrane proteins and IDPs, where sequence-structure relationships diverge from globular paradigms (Agarwal and McShan, 2024).

### 4.2 Convolutional neural networks

A convolutional neural network (CNN) is a type of neural network. It is a feed-forward neural network with a deep structure and convolutional calculation. The structure of the CNN is shown in Figure 3b. The amino acid sequences are converted to a two-dimensional matrix as input after being represented by solo thermal encoding or embedding and pre-processed using strategies such as normalization, and principal components analysis. It then enters the convolution layer, where the convolutional operations extract features, enhance signal characteristics, and reduce noise. Following this, after pooling, the data features remain unchanged while the data is compressed, thereby reducing overfitting. After several rounds of convolution and pooling operations, the input data is abstracted into features with higher information content. These then enter the fully connected layer to generate prediction results based on the final extracted data features.

Gabriel Cretin et al., leveraging the capabilities of deep neural networks, proposed the PYTHIA method. This approach incorporates a deep residual incidence neural network with a convolutional block attention module to predict the local conformation of a protein directly from the amino acid sequence Cretin et al. (2021). TBM, which aims to construct structural models by replicating and refining the structural framework of other known proteins, is an accurate method for protein structure prediction. However, it is challenging to identify distant homologous templates, and as a result, the accuracy of TBM rapidly decreases when the evolutionary relationship between the target and the template diminishes. Lupeng Kong et al. proposed a novel deep learning method, named ProALIGN, that predicts accurate sequence–template comparisons (Kong et al., 2022). Protein alignment are represented as a binary matrix, after which a deep convolutional neural network is employed to predict the optimal permutation from the query protein and its template. This method can enhance the accuracy of matching target proteins from the TBM method, with the template proteins in the protein database. This improves subsequent protein structure prediction and enhances the overall accuracy of protein structure prediction. Protein secondary structure prediction is crucial for studying protein structure and function. Both traditional machine learning methods and deep learning neural networks have been utilized, and have made great progress in approaching the theoretical limits. Shiyang Long et al. constructed a contextual convolutional neural network (Contextnet) with high accuracy on the JPred and CASP13 datasets (Long and Tian, 2019). The CNN also successfully integrated 1D structural features, 2D contact information, and 3D structural quality scores to improve protein model quality assessment, where contact prediction using convolutional neural networks was first shown to consistently improve protein model rankings. Convolutional neural networks can predict tertiary structures directly from protein as well as structural sequences, and Timmons et al. proposed the use of neural networks and simulated annealing algorithms to predict tertiary sequences from peptide primary sequences to help accelerate the peptide drug design process (Timmons and Hewage, 2021). A large convolutional residual neural network proposed by Jinbo Xu et al. can predict the correctly folded structures of 26 of the 32 free model targets of CASP13 and L/5 long-range contacts with an accuracy of over 80% Xu J. et al. (2021a).

### 4.3 Recurrent neural networks

Recurrent Neural Networks (RNNs) are a class of neural networks designed to handle sequential data, performing recursive operations along the sequence’s evolution direction. All nodes (recurrent units) are connected in a chain-like manner. The RNN can also be expanded vertically by increasing the number of layers of the network as in other neural networks, as shown in Figure 3c. Long short-term memory (LSTM) is a temporal recurrent neural network designed to solve the long-term dependency problem of general RNNs, all of which have a chained form of repeating neural network modules. A recurrent unit is a type of RNN. Like LSTM, it was proposed to solve problems such as long-term memory and gradients in back propagation. The LSTM model is shown in Figure 3d. A GRU is a simple variant of LSTM. It is simpler in structure, is no less effective, and is more efficient in operation than LSTM networks, making it a popular network structure at present. The GRU can achieve comparable results, which can improve the training efficiency to a great extent. Its structure is shown in Figure 3e. Proteins exhibit strong sequential characteristics at the primary structure level, and models such as RNN, LSTM, and GRU can predict their 3D tertiary structures based on this sequence information.

Protein secondary structure provides crucial structural insights, and its accurate prediction from primary sequences is pivotal in protein research. The local interactions and neighboring residues in the primary sequence determine the secondary structure formation. RNNs, LSTM networks, and GRUs have demonstrated remarkable performance in predicting protein secondary structures from amino acid residue information and capturing long-range interactions. Yanbu Guo et al. proposed 2D convolutional bidirectional recurrent neural networks (2C-BRNNs) (Guo et al., 2018) to improve the accuracy of secondary structure prediction by extracting discriminative local interactions between amino acid residues and then further capturing the interactions between amino acid residues using bidirectional gated recurrent units or bidirectional LSTM. AK Sharma et al. proposed the use of deep RNNs to predict the secondary structure of proteins from primary sequences. Bidirectional LSTM models (Sharma and Srivastava, 2021) have been used to extract past and unknown residue information from primary sequences, on which the description and understanding of protein structure rely heavily. In protein NMR studies, it is more convenient to predict the secondary structure from chemical shifts than from inter-nuclear distances. Zhiwei Miao et al. proposed a deep neural network based on bi-directional LSTM (Miao et al., 2021) to predict the 3-state secondary structure of proteins using the NMR chemical shifts of the backbone. Wei Zhong et al. proposed clustered RNNs as a method for protein tertiary structure prediction (Zhong and Gu, 2020) using RNNs from multiple sample clusters organized in a hierarchical tree structure to learn local sequence–structure relationships at different granularity levels. Their model can learn the non-linear sequence–structure relationships of proteins more effectively than a single machine learning model. Understanding protein sequence–structure relationships is key to using sequence information to predict the 3D structure of proteins. J Antony et al. combined LSTM and bidirectional LSTM neural network architectures for predicting the tertiary structure of proteins from primary sequences (Antony et al., 2021), and their results showed that bidirectional LSTM networks with primary sequence and site-specific scoring matrix data as input had high accuracy. Lina Yang et al. were able to better handle long sequences by building a GRU neural network that can handle long sequences for learning long-term dependencies well (Yang L. et al., 2020). They combined batch normalization with GRU to construct a new network, and a position-specific scoring matrix was used to correlate with other features to build a completely new feature set, thus effectively improving prediction accuracy.

### 4.4 Graph neural networks

Graph neural networks (GNNs) have become a research hotspot in areas such as natural language processing, computer vision, and traffic prediction. Graph convolutional networks have shown practical utility in the field of bioinformatics. The protein backbone holds proteins together and produces the tertiary structure of a protein. The amino acid sequence of a protein is used as input to predict the geometry of the 3D protein backbone structure. This is a sequence-to-structure prediction task that abstracts the protein structure to that of a graph, extracting the features of the nodes and edges, in the process shown in Figure 3f.

Determining the three dimensions of a protein from its sequence is one of the most challenging problems in biology. Geometric deep learning has been highly successful in the fields of social networking, chemistry, and computer graphics. Although it is natural to render protein structures as 3D shapes, few existing studies have examined protein structures directly as graphs. Tian Xia et al. explored the geometric deep learning and proposed a graphical neural network architecture to address these challenges (Xia and Ku, 2021). The proposed protein geometric GNN models distance geometric representations and dihedral geometric representations by geometric graphical convolution. This study shed new light on the study of protein 3D structures. The authors validated the effectiveness of GNNs on multiple datasets. AlphaFold2 and related systems use deep learning to predict protein structures from co-evolutionary relationships encoded in MSAs. Despite recent dramatic improvements in accuracy, the following challenges remain: (i) predicting proteins that cannot generate MSAs templates and that evolve rapidly; (ii) rapidly exploring designed structures; and (iii) understanding the rules of spontaneous polypeptide folding in solution. Ratul Chowdhury et al. reported the development of an end-to-end distinguishable recursive geometric network (RGN) that can predict protein structures without using MSAs from an individual protein sequence to predict protein structure (Chowdhury et al., 2022). Compared to AlphaFold2, the RGN is superior in predicting distal protein structures. The prediction of protein secondary structure based on amino acids is important for gathering information about protein features and their mechanisms, such as the catalytic function of enzymes, biochemical reactions, and DNA replication. Tamzid Hasan Nahid et al. proposed a new technique for predicting protein secondary structure using GNNs Nahid et al. (2021). First, a graph is drawn from a dataset using primary sequences (amino acids). The entire graph is then iterated sequentially using a GNN to summarize the information of neighboring nodes. The method has high accuracy in the prediction of eight states of protein secondary structure.

### 4.5 Deep residual neural networks

Deep learning networks can improve the learning efficiency by increasing the number of layers, but the classification and recognition prediction of deeper networks are not improved by increasing the number of layers. Rather, the gradient disappears due to the stacking of layers. Deep residual neural networks can deepen the network and solve the gradient disappearance problem at the same time. Figure 3g shows the protein 3D structure predicted by ResNet after inputting the template structure and query sequence to the feature tensors.

Protein structure prediction (PSP) is considered to be a complex problem in computational biology. Although co-evolution-based approaches have made significant progress in PSP, it is still a challenging and unsolved problem. Predicting contacts and distances between residues from co-evolutionary data using deep learning has greatly advanced protein structure prediction (Wu and Xu, 2021). F Wu et al. proposed a new method, New Deep Learning Threader (ND Threader), to refine sequence–template alignments from predicted protein distances. It is a good premise for protein structure prediction. The method is based on TBM and uses an integration of deep ResNet (residual neural network) and conditional random field to align query proteins to templates without using any distance information. The sequence–template alignment and input to deep ResNet were then used to predict the interatomic distance distribution, and a 3D model was constructed using PyRosetta. A deep residual network was developed by Jianyi Yang et al. to predict the direction of residuals in addition to distance (Yang et al., 2020a). This model assigned higher probabilities to newly designed proteins and helped identify the key residues that determine folding. The method is expected to be used for a wide range of protein structure prediction and design problems. S. Geethu et al. proposed a new method for predicting inter-residue distances and dihedral angles using a deep ResNet architecture designed to generate an average of 125 homologous sequences from a set of custom sequence databases (Geethu and Vimina, 2021). These sequences were used to generate input features. As a result of the neural network, a structure library was generated, from which the lowest potential structure was selected as the final predicted 3D protein structure. H Zhang et al. showed that a new TBM approach, called ThreaderAI (Zhang and Shen, 2020), improved protein tertiary structure prediction. ThreaderAI formulated the task of querying sequence to template alignment as a computer vision and a classical pixel classification problem and applied deep residual neural networks for the prediction. ThreaderAI first uses deep learning to predict the probability matrix of residue–residue alignment by integrating sequence profiles, predicted sequence structural features, and predicted residue–residue contacts and then builds a template–query alignment by applying a dynamic programming algorithm to the probability matrix using the aligned template for structure prediction with high accuracy.

### 4.6 Transformer

The transformer is a deep learning architecture that has gained widespread popularity in natural language processing tasks, particularly in the context of machine translation. The key innovation of the transformer is its ability to capture long-range dependencies between input sequences, which is particularly relevant in the case of protein sequences, where long-range interactions between amino acids are critical to determining the final structure (Jiang et al., 2023). The multi-head attention mechanism, on the other hand, enables the model to attend to different parts of the sequence simultaneously, allowing it to capture both local and global features of the protein. For example, Alphafold2, the researchers utilized a combination of the transformer architecture and multi-head attention mechanism, along with other innovations such as the use of distance constraints and evolutionary information, to predict protein structures with unprecedented accuracy. Similarly, other models such as RosettaFold and ESMfold have also incorporated the transformer architecture and multi-head attention mechanism, with impressive results. The quality of the input MSAs is therefore a key factor in determining whether a high-accuracy model can be produced. DMFold algorithm (Zheng et al., 2024), which excelled in the protein complex structure prediction category of the recent CASP15 competition by integrating DeepMSA2 with the state-of-the-art AlphaFold2 modeling approach. Compared with existing MSA construction methods, one of the major advantages of DeepMSA2 lies in the iterative search and model-based preselection strategy, which can result in MSAs with more balanced alignment coverage and homologous diversity. The 2024 release of AlphaFold3 by DeepMind Abramson et al., 2024) represents a revolutionary breakthrough in biomolecular structure prediction. Unlike its predecessor AlphaFold2 which focused solely on protein structures, AlphaFold3 achieves end-to-end joint prediction of proteins, nucleic acids (DNA/RNA), small molecule ligands, and their complexes. The architecture replaces AlphaFold2’s Evoformer with a Pairformer module, reducing reliance on multiple sequence alignments (MSAs) while improving data utilization efficiency. The framework introduces a geometric diffusion model that enables probabilistic sampling of complex conformations, significantly enhancing the modeling capability for flexible interfaces and allosteric effects, thereby extending its applicability to a broader range of biomolecules.

### 4.7 Diffusion-based model

Although AlphaFold2 and alternative models such as RoseTTAFold Baek et al. (2021), ESMFold Lin et al. (2022), and OmegaFold (Wu et al., 2022) are widely considered to have successfully addressed the challenge of predicting protein structures from sequences, their effectiveness is primarily limited to globular proteins with a clear counterpart or homologous crystallized protein in the training dataset (the PDB). These models are developed and trained as deterministic mappings from input (sequence or MSA) to output (structure), which limits their ability to model structural ensembles. As generative models, diffusion-based models learn an iterative, stochastic generative process that model multimodal data distributions and generate samples efficiently (Watson et al., 2022), applied in many domains, including molecules generation (Hoogeboom et al., 2022), protein-ligand complex structure generation (Nakata et al., 2023) and protein structure generation (Anand and Achim, 2022; Trippe et al., 2022; Wu et al., 2024; Fu et al., 2023). There have been a limited number of diffusion models designed for forward problems involving protein structures (Nakata et al., 2023; Qiao et al., 2022). However, a recent study firstly designed a diffusion generative modeling framework (called EignFold) for protein structure prediction from a fixed protein sequence. EignFold (Jing et al., 2023) is a novel harmonic diffusion process that models the molecule as a system of harmonic oscillators and explored the application of diffusion modeling to protein structural ensembles, aiming to develop a tool for modern structure prediction frameworks.

### 4.8 Large language model

Large language models (LLMs) are built on a transformer with many parameters which enable the model to better understand the relationships between different elements of the input Høie et al. (2022). They have recently applied to machine translation, question answering, language-image pre-training with emerging functionalities, such as performing higher-level reasoning and generate lifelike images and text. Recent advances have proved the power of large language models in processing the protein sequence databases (Lin Z. et al., 2023; Wu et al., 2022; Fang et al., 2022). The primary, secondary, tertiary, and quaternary of protein structures bear an analogy to the letters, words, sentences, and texts of human language Hu et al. (2022). These characteristics of reused and rearranged of modular elements significantly benefit the development of protein large-scale language models. For example, Meta AI, FAIR Team developed a high accuracy end-to-end atomic level protein structure prediction method using the individual sequence of a protein, called ESMFold. ESMFold has up to 15 billion parameters and is the largest protein language model to date (Lin et al., 2022). Different from the AlphaFold2, RoseTTAFold and other related models that use deep learning and MSAs (Jumper et al., 2021; Yang et al., 2020a; Baek et al., 2021), Chowdhury et al., proposed an end-to-end protein language model (named AminoBERT) (Chowdhury et al., 2022) using single protein sequences. These protein large language models open up new possibilities for protein structure prediction, especially proteins that have not been structurally characterized before.

### 4.9 Applications in disease-related protein structure prediction

Deep learning methods for protein structure prediction have demonstrated significant practical value in addressing urgent public health challenges. A notable example is the application of AlphaFold during the COVID-19 pandemic. In early 2020, when SARS-CoV-2 was first emerging, DeepMind rapidly deployed AlphaFold to predict structures of several understudied viral proteins, including the membrane protein, protein 3a, Nsp2, Nsp4, Nsp6, and Papain-like proteinaseTeam (2020). These predictions were released to the scientific community before experimental structures were available, providing valuable insights for understanding viral mechanisms and accelerating therapeutic development. The accuracy of these predictions was later validated when the experimental structure of ORF3a protein was determined, confirming AlphaFold’s ability to predict novel protein folds accurately. As the pandemic evolved, deep learning methods continued to provide crucial insights into new variants. Yang et al. utilized AlphaFold2 to predict the structures of S, M, and N proteins in the Omicron variant, with particular emphasis on analyzing the structural alterations in the RBD and NTD regions of the S protein and their potential implications for viral transmission and immune evasion. This study provided crucial structural insights for the development of vaccines and therapeutic strategies targeting the Omicron variant (Yang et al., 2021). More recent applications have extended to other emerging diseases. For instance, Sahu et al. employed RoseTTAFold to predict protein structures of Monkeypox virus targets. By combining these structural predictions with computational drug screening, they identified potential FDA-approved drugs that could be repurposed to target these viral proteins (Sahu et al., 2023). This approach demonstrates how AI-powered structure prediction can accelerate the drug discovery process by enabling rapid identification of therapeutic candidates for emerging diseases. These applications highlight how deep learning methods have transformed from purely academic tools into practical solutions for urgent public health challenges, enabling rapid response to emerging diseases through structure-based drug discovery and therapeutic development.

## 5 Model validation

Deep learning models are often evaluated by cross-validation (Berrar, 2019; Liu et al., 2021), in which the original observation dataset is divided into a training set for model training and a separate set for evaluating model performance (Zhong and Gu, 2020; Cretin et al., 2021; Akbar et al., 2021; Xu et al., 2020). The most commonly used cross-validation methods include hold-out cross-validation (Ziggah et al., 2019), k-fold cross-validation (Wong and Yeh, 2019), and leave-one-out cross-validation (LOOCV) Magnusson et al. (2020). Hold-out cross-validation splits the dataset into two mutually exclusive sets; that is, the training and test sets have no cross-over samples. This requires that the number of samples in the training set is at least 50% of the total number of samples. However, there are limitations to hold-out cross-validation, as this validation method only performs one division, and when the division of the dataset is not performed randomly, the evaluation results are subject to chance. This can lead to underfitting or overfitting when the training and test sets are not evenly distributed. K-fold cross-validation is a widely used cross-validation technique. It divides the dataset into k equally-sized, mutually exclusive sets at random. Then, the k sets are used as the test set and the rest as the training set, and the final validation result is averaged after k validations. As each data appear once in the validation set and k-1 times in the training set, this will significantly reduce underfitting. The majority of the data in the dataset is used for training, and the possibility of overfitting is also reduced. LOOCV is a special type of k-fold cross-validation. In LOOCV, the value of k is the number of samples in the dataset. One sample at a time is used as the test set and the rest as the training set, which provides the closest expectation to training on the entire test set. LOOCV, being the most objective method, is therefore used by many researchers to test the ability of various prediction methods. However, when the number of proteins in a given set is not large enough, the sequential exclusion of each protein from the set may result in a severe loss of information. In such cases, the leave-one-out test cannot be utilized.

Q3 accuracy and Q8 accuracy are among the most frequently used evaluation metrics by researchers in protein secondary structure prediction Drori et al. (2018), protein structures are diverse, but the torsion angles and hydrogen bonds in protein structures are repetitive, allowing the classification of protein residues into relatively few structural categories. In the 1980s, the Dictionary of Secondary Structure Patterns (DSSP) proposed eight residue categories, which were later combined into three categories in order to ease the difficulty of protein structure prediction.

$$Q_{m}=\frac{100\times \sum_{i=1}^{N_{\text{res}}}M_{i}}{N_{\text{res}}}$$ (1)

where m = 3 and m = 8 is referred as $Q_{3}$ and $Q_{8}$ accuracy, respectively. $N_{\mathrm{res}}$ is the total number of residues, and $M_{i}$ is correctly predicted number of residues in state i Equation 1. Thus, Q8 and Q3 provide the overall percentage of trimers and octamers that have their residues correctly predicted. The root mean square deviation (RMSD) Maiorov and Crippen (1994) is a traditional and commonly used metric for assessing the quality of predicted structures.

$$D\left(A,B\right)=\sqrt{\frac{1}{n\left(n-1\right)/2}\underset{i<j}{\sum }{\left(d_{ij}^{A}-d_{ij}^{B}\right)}^{2}}$$ (2)

where n is the number of atoms in protein, $d_{ij}^{A}$ and $d_{ij}^{B}$ are the corresponding distances between the ith and jth atoms Equation 2. The RMSD calculates the average distance between equivalent atom pairs in two best stacked protein structures. Typically, only backbone atoms are involved in the RMSD calculation.

TM-score (Zhang and Skolnick, 2005) is a metric for assessing the topological similarity of protein structures. TM-score weights smaller distance errors more heavily than larger distance errors, making the score values more sensitive to global folding similarity compared to local structural variation.

$$\text{TM}-\text{score}=\max\left\{\frac{1}{L_{\text{target}}}\overset{L_{\text{aligned}}}{\underset{i=1}{\sum }}\frac{1}{1+{\left(\frac{d_{i}}{d_{0}\left(L_{\text{target}}\right)}\right)}^{2}}\right\}$$ (3)

Here, $L_{\text{aligned}}$ is the number of residues in the aligned regions, $L_{\text{target}}$ is the length of the target protein, $d_{i}$ is the distance between corresponding residues in the target and predicted structures, and $d_{0}\left(L_{\text{target}}\right)$ is a normalization factor adjusted based on the target protein’s length, facilitating comparisons across proteins of different sizes Equation 3. The TM-score introduces a length-dependent scale to normalize the distance error, making the size of the TM-score independent of the length of the random structure pair, thus allowing the TM-score to refine traditional metrics such as RMSD. The Global Distance Test (GDT-score) is calculated based on the largest set of residue-residue pairs that fall within a defined distance from the demarcation line given the superimposed structure (Chen and Cheng, 2022). The Global Distance Test Total Score (GDT_TS) (Li et al., 2016) is a threshold-based measure that determines the topology.

$$\text{GDT}-\text{TS}=\frac{1}{4}\left({\text{GDT}}_{1}+{\text{GDT}}_{2}+{\text{GDT}}_{3}+{\text{GDT}}_{4}\right)$$ (4)

Each ${\text{GDT}}_{i}$ score is calculated as the percentage of Cα atoms in the predicted structure that are within the corresponding distance threshold from the native structure, multiplied by 100. ${\text{GDT}}_{1}$ , ${\text{GDT}}_{2}$ , ${\text{GDT}}_{3}$ and ${\text{GDT}}_{4}$ are the scores for the 1.00Å, 2.00Å, 3.00Å, and 4.00Å thresholds, respectively Equation 4. GDT_TS is the most widely used scoring method to assess the overall quality of a model after CASP4. GDT scores typically range from 0 to 100, with higher scores indicating a more perfectly constructed target backbone conformation. The metric also shows a strong dependence on protein length. GDT_TS allows comparison of results within and between experiments, and this focus on similarity allows the measure to distinguish models that are poor, but contain locally correct fragments, from those that are globally wrong in a way that other related measures cannot.

In recent years, researchers have developed numerous quality assessment (QA) methods to evaluate the accuracy of predictions for protein quaternary structures. One widely used metric is LDDT/pLDDT (Local Distance Difference Test/Probability Local Distance Difference Test), which measures the local structural similarity between predicted and native structures. This metric calculates the difference between predicted and native structures at each residue and averages these differences over the entire structure. LDDT/pLDDT has proven effective in evaluating the accuracy of predicted protein structures, especially when experimental data is unavailable. Another metric, DockQ Score, is specifically designed to evaluate the accuracy of predicted protein-protein docking structures. This composite score considers various aspects of the predicted structure, including shape complementarity, electrostatics, and desolvation energy. DockQ Score has also demonstrated effectiveness in evaluating the accuracy of predicted protein-protein docking structures when compared to other available metrics. For example, studies such as Chen et al. (2022) and Chen et al. (2023) have utilized these metrics to assess protein structure predictions.

## 6 Conclusion

**FIGURE 4:**
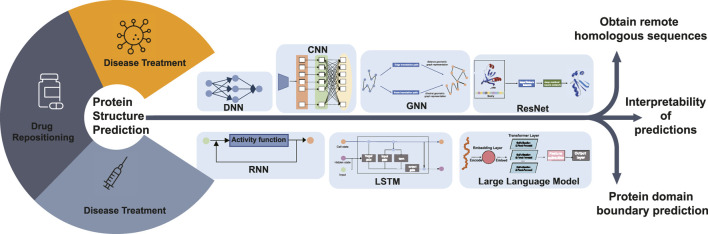
Diagram illustrating the future research hotspots and application scope of deep learning-based protein structure prediction. Protein structure prediction is the basis for disease diagnosis, drug repositioning, and vaccine development research. Future research can predict the 3D structure of proteins, including obtaining remote homologous sequences, interpretability of protein structure predictions, and protein domain boundary prediction, by DNN, CNN, GNN, RNN, LSTM, ResNet, and LLM deep learning algorithms.

Protein structure prediction has a crucial role in bioinformatics because protein structure determines protein function. The study of protein structure is fundamental to research areas such as drug repositioning, disease treatment, and protein function. The study of protein structure prediction has received increasing attention from researchers with the continuous development of deep learning techniques and especially the use of deep learning models to accomplish protein structure prediction tasks. This paper provides a summary of protein structure prediction based on deep learning, and it is easy to see that the addition of deep learning methods has made a significant contribution to protein structure prediction. As shown in Figure 4, in the coming years, we are likely to see more advances in protein structure prediction.

For template modeling, all protein structure prediction methods take multiple sequence alignment as input. Therefore, access to homologous sequences becomes the main reason for improved prediction results. Searching for remote homologous sequences in databases can be a difficult research problem. Finding sequence information in major sequence databases to generate multiple sequence comparisons and using more powerful search algorithms to generate multiple sequence comparisons quickly offer possibilities in this field.

Deep learning models are often considered black-box systems because the internal decision-making processes are not easily interpretable to humans. While the models consistently make predictions, the intricate workings of how input data is transformed into predictions can be complex and not straightforward to understand. This is also true in bioinformatics, so research into the interpretability of deep learning models could enhance the interpretability of the sequence-to-structure process in protein structure prediction. In addition, the sequence–structure–function relationship of proteins can be complemented and refined.

The task of single-domain protein structure prediction has been accomplished to some extent, and the correct assignment of domain boundaries from sequences is a key step toward accurate multi-domain protein structure prediction. Future work could be carried out in the area of deep learning-based prediction of multi-domain protein structures. Zheng et al. (2021) developed a contact-based domain boundary prediction algorithm, FUpred, for detecting protein domain boundaries, which could be a new trend in protein structure research. The majority of protein structure prediction methods, including AlphaFold2, focus primarily on predicting static protein structures, which is a significant limitation, but proteins undergo conformational changes and flexible motions under physiological conditions, which are important for their functions. As a unified structure prediction tool, AlphaFold3 has significantly expanded its prediction scope, capable of not only predicting protein structures but also the structures and interactions of various biomolecules including DNA, RNA, antibodies, small molecules. While achieving such comprehensive coverage, AlphaFold3 has also improved prediction accuracy, particularly demonstrating significant advances in predicting protein-DNA complexes and protein-antibody complexes. However, AlphaFold3 still faces certain limitations. Approximately 4.4% of predictions exhibit chirality mismatches or steric clashes, particularly prominent in large complexes. Furthermore, similar to its predecessors, AlphaFold3 primarily focuses on predicting static conformations and may not fully capture protein dynamic transitions. These challenges indicate that despite major breakthroughs in deep learning-based structure prediction, there remains substantial room for improvement in enhancing prediction accuracy and molecular dynamics simulation. Structure prediction is only the first step. Challenges remain in using these structures for better functional annotation and designing new proteins. In summary, despite the breakthrough, there is still ample room for improvement in protein structure prediction, such as handling complex cases, capturing dynamic features, improving time efficiency, and reducing reliance on experimental data. Future efforts are needed to address these challenges and push the boundaries of this field.

With the advancing development of protein structure prediction techniques, they will play an even more important role in biology and medicine. For example, in the development of vaccines, proteins act as scaffolds for immunogens. In disease treatment, proteins act as receptors that bind to drugs for pharmacological responses and act as drug carriers that integrate multiple targeting cues. Proteins are designed to make drugs active in specific environments to reduce side effects. The importance of protein structure prediction is reflected in the need for protein structures to support research in all of these application areas. As protein structure data grow exponentially and provide a larger platform for protein structure prediction, the possibility of using these data to create new methodological techniques is opened.

## 7 Key points

• A comprehensive review and summary of datasets involved in protein structure prediction, providing an up-to-date overview of available resources in this field.

• Protein structure prediction based on deep learning has been receiving increasing attention. These approaches can capture underlying features and grasp the complex structures of amino acid sequence information.

• Evaluation metrics are clearly listed for the evaluation of computational protein structure prediction model.

• There are several challenges to future trends for protein structure prediction, including homologous sequences generation, interpretable deep learning approaches and automation pipeline.
